# Macrophage Heterogeneity in Kidney Injury and Fibrosis

**DOI:** 10.3389/fimmu.2021.681748

**Published:** 2021-05-20

**Authors:** Yi Wen, Hong-Ru Yan, Bin Wang, Bi-Cheng Liu

**Affiliations:** Department of Nephrology, Zhongda Hospital, Southeast University School of Medicine, Nanjing, China

**Keywords:** macrophage heterogeneity, resident macrophage, kidney, inflammation, fibrosis

## Abstract

Kidney macrophages are central in kidney disease pathogenesis and have therapeutic potential in preventing tissue injury and fibrosis. Recent studies highlighted that kidney macrophages are notably heterogeneous immune cells that fulfill opposing functions such as clearing deposited pathogens, maintaining immune tolerance, initiating and regulating inflammatory responses, promoting kidney fibrosis, and degrading the extracellular matrix. Macrophage origins can partially explain macrophage heterogeneity in the kidneys. Circulating Ly6C^+^ monocytes are recruited to inflammatory sites by chemokines, while self-renewed kidney resident macrophages contribute to kidney repair and fibrosis. The proliferation of resident macrophages or infiltrating monocytes provides an alternative explanation of macrophage accumulation after kidney injury. In addition, dynamic Ly6C expression on infiltrating monocytes accompanies functional changes in handling kidney inflammation and fibrosis. Mechanisms underlying kidney macrophage heterogeneity, either by recruiting monocyte subpopulations, regulating macrophage polarization, or impacting distinctive macrophage functions, may help develop macrophage-targeted therapies for kidney diseases.

## Introduction

Macrophage plays an important role in kidney disease pathogenesis and is a potential therapeutic target for kidney injury and fibrosis. Kidney macrophage subpopulations can either promote or prevent the extracellular matrix deposition in the kidney, drawing the possibility of reversing kidney fibrosis (1). However, the functionally opposing macrophage subpopulations rising ambivalence in understanding macrophage activities during kidney injury and fibrosis, disturbing the development of macrophage-targeted therapies (2). Studies have focused on macrophage functional diversities mechanisms and applied novel approaches in precisely identifying macrophage subpopulations. Therefore, intriguing questions have arisen, such as the macrophage origin (kidney-resident macrophages vs. circulating monocyte precursors), macrophage differentiation (oversimplified M1/M2 categorization vs. newly subsets defined by cell surface markers and single-cell RNA-sequence), and their effector functions in the pathogenesis of kidney diseases.

## Macrophage Heterogeneity

Macrophage heterogeneity attracts attention since the discovery of macrophages. Following studies expand macrophage heterogeneity definition depending on the origin, cell surface markers, and cytokines secretion (3). Macrophages obtain distinct phenotypes under physiological conditions and differentiate into functional phenotypes in response to pathological stimulation. According to their cooperation with distinct T cell subsets, macrophages have generally been classified either into classical M1 or alternative M2 macrophages. M1 macrophages are characterized by pro-inflammatory effects and engage with T helper 1 (Th1) cells, whereas M2 macrophages exhibit immunoregulatory efforts and intimately cooperate with T helper 2 (Th2) cells (4). M1 macrophage differentiation is initiated by pathogen-associated molecular patterns (PAMPs), danger-associated molecular patterns (DAMPs), and pro-inflammatory cytokines, especially under acute deleterious conditions (5). Representative functions of M1 macrophages are host defense and secretion of pro-inflammatory cytokines, such as tumor necrosis factor-α (TNF-α), interleukine-1β (IL-1β), interleukine-6 (IL-6), and interleukine-12 (IL-12). M2 macrophage is typically polarized by interleukine-4 (IL-4) and interleukine-13 (IL-13), suppressing inflammation and promoting wound repair. Recent studies further classify M2 macrophages into different subsets depending on their differentiation stimuli, markers, and functions (6). While classic M2 macrophages are classified into M2a macrophages, M2b macrophages are induced by immune complexes, toll-like receptors and/or interleukine-1 receptors (IL-1R), contributing to immunoregulation and Th2 cells activation. M2c macrophages are induced by interleukine-10 (IL-10) and glucocorticoids, participating in immunosuppression, tissue repair, and matrix deposition. The simplified paradigm of M1/M2 macrophages facilitates the studies of macrophage phenotypes and functions. However, typical M1/M2 macrophages are induced in carefully regulated circumstances *in vitro* and not uniformly observed under the dynamic and complex environment *in vivo*. In fact, macrophages appear to express M1/M2 markers simultaneously during kidney injury, and their origins largely determine the functions of renal macrophages. Lineage tracing studies demonstrated that postnatal kidney macrophages predominately originate from the yolk sac EMP and hematopoietic stem cells, and bone marrow-derived monocytes infiltrate the kidney under inflammatory conditions (7, 8).

## Renal Macrophage Distribution During Development, Health, and Disease

Macrophages contribute to morphogenesis during organ development. In cultured kidney explants, colony-stimulating factor-1 (CSF-1) application stimulates ureteric bud branching and nephron formation (9). By binding to the membrane receptor CSF1R, CSF-1 accelerates macrophage proliferation and differentiation (10, 11). Macrophages infiltrate the nephrogenic zone and facilitate nephron progenitor proliferation after the transient loss of nephron progenitor cells (12). Munro et al. (13) demonstrated that macrophages directly interacted with endothelium in developing cortical nephrogenic caliber vessels. These F4/80^+^CD206^+^ macrophages are perivascular and enriched for mRNAs associated with organogenesis. Moreover, the subpopulation of Gal3^high^ myeloid cells are professional phagocytes and intermingle with pro-development F4/80^+^CD206^+^ macrophages in the developing mouse kidney. Therefore, fetal kidney macrophages possibly facilitate organogenesis by interacting with newly forming nephrogenic blood vessels. In addition, macrophages distribute around renal tubules during kidney development (9), but the underlying mechanisms are unclear. Experimental models of invertebrate species, such as *Drosophila melanogaster* (*D. melanogaster*), provide novel insights. In *D. melanogaster*, the Malpighian tubules are analogous to kidneys, while hemocytes are similar to macrophages. Hemocyte deposition around the developing Malpighian tubules is mediated by type IV collagen, necessary for the normal organogenesis of anterior Malpighian tubules (14). In mammalian kidneys, the renal tubular basement membrane is abundant in collagen IV and attracts macrophage recruitment (15, 16). However, it is difficult to extend these findings to mammalian kidneys due to the unique nephron structure.

In normal human kidneys, Marshall et al. (17) observed monocyte/macrophage distribution by immunoperoxidase staining of α-1-antitrypsin, muramidase, and serum 22. Most positive staining cells were scattered in glomerular capillaries and intertubular blood vessels. Macrophages rarely infiltrated into tubulointerstitium except in scarring tissues. However, the generality of these findings is limited by non-specific markers. Recently, Cao et al. (18) demonstrated that F4/80^+^CD11c^−^ macrophages distribute throughout the renal cortex and medulla in healthy kidneys. In contrast, F4/80^+^CD11c^+^ mononuclear phagocytes are distributed in the renal cortex rather than the medulla under normal and injured conditions. These double-positive mononuclear phagocytes performed M1-like macrophage phenotype and aggravated kidney injury during Adriamycin nephropathy. However, a large percentage of CD11c^+^ dendritic cells co-express F4/80 marker in healthy kidneys, the preference to determine the nature of F4/80^+^CD11c^+^ cells should be circumspect.

Due to a double set of arterioles and capillaries, the kidney owns a unique vascular supply and receives about 25% of the cardiac output. The renal cortex exposed continuously to large amounts of blood-derived antigens and antibodies, resulting in a high sensitivity to renal glomerular diseases (19). Glomerular macrophage accumulation is an important feature in human glomerulonephritis. Macrophage clearance decreased glomerular damage in experimental glomerulonephritis (20). Further studies targeting monocyte chemotactic molecule-1 (MCP-1) or leukocyte adhesion molecules (ICAM) successfully attenuated macrophage accumulation and kidney injury in experimental models (21, 22). Interestingly, the numbers of tubulointerstitial macrophages rather than glomerular macrophages predict renal dysfunction (23, 24). Our previous studies found that tubular epithelial exosomes contribute to macrophage infiltration and activation, providing a novel insight into tubulointerstitial macrophages (25–27). This review focus on macrophage heterogeneity in the kidney and excellent works have been done to card macrophage function and distribution during acute kidney injury (AKI) and chronic kidney disease (CKD) (1, 28).

## Kidney Resident Macrophage Origin and Specificity

Kidney-resident macrophages are *in situ* self-renewed and characterized by their phagocytic activities, expression of pattern recognition receptors (PRRs), and immunological regulation capacity, thus, maintaining kidney homeostasis (29). Kidney-resident macrophages derive from 3 sources: (1) yolk sac erythro-myeloid progenitors (EMP)-derived macrophages, (2) fetal liver EMP-derived macrophages, and (3) hematopoietic stem cells (HSC)-derived macrophages. The relative proportion of each progenitor dramatically changes during the development, adulthood, and damaged kidney state.

At embryonic day 12.5, kidney macrophages are CD45^+^ CD11b^lo^ F4/80^hi^ Ly6C^−^ cells deriving from yolk sac EMP; in contrast, CD45^+^ CD11b^hi^ F4/80^lo^ Ly6C^+^ monocytes are undetectable at this stage (30). Using tamoxifen-inducible Runx1^Cre/EYFP^ and Csf1r^Cre/EYFP^ mice, fate tracing studies demonstrated that the relative proportion of yolk sac EMP-derived macrophages in the kidney decrease dramatically after embryonic day 13.5 (30). Consistently, *Csf1r-Cre* positive yolk sac-derived macrophages represent a minimal proportion of kidney macrophages after postnatal, possibly due to their dilution by the later arrival of fetal liver EMP-derived and HSC-derived macrophages (7). Sheng et al. (8) provided evidence of HSC-derived kidney macrophages using tamoxifen-inducible c-Kit^Cre/EYFP^ mice. They further concluded that HSC precursors rather than EMPs are the source of kidney resident macrophages (8). However, the non-specific expression of *c-Kit-Cre* makes this conclusion debatable (31, 32). As HSCs transiently expressing *Flt3-cre (* 33), Epelman et al. (7) distinguished the origin of HSC-derived monocytes and EMP-derived monocytes using Flt3^Cre/GFP^ mice and found their equal contribution to the pool of resident macrophages. In contrast, Hoeffel et al. (30) demonstrated that fetal liver EMP-derived c-Myb^+^ monocytes are the predominant source of kidney resident macrophages. Thus, further studies based on fate-mapping studies must concern the limitations of genetic models, and single-cell RNA-sequence classifies ability worth more attention. While the kidney exposes to circulating monocytes throughout the development and adulthood, the kidney resident macrophages are mainly EMP- and HSC-derived rather than bone marrow-derived (7, 8, 30, 32, 34), partially explained by the niche competition hypothesis (35). Recent studies found that under certain types of kidney disease, expanded macrophages derive from the subset of resident macrophages, especially yolk sac-derived macrophages (36, 37). Ide et al. (37) demonstrated that CX3CR1^+^ yolk sac-derived macrophages have a higher proliferating capacity and progressively expand in number in older mice kidneys. Kidney resident macrophage proliferation contributes to the proangiogenic and pro-inflammatory environment after ischemic AKI and is confirmed by staining with Ki67 or BrdU (36, 38).

Kidney resident macrophages monitor trans-endothelial transport of circulating immune complexes and regulate the infiltration of lymphocytes and neutrophils (39). Using an unbiased flow cytometry approach, Kawakami et al. (40) classified kidney resident mononuclear phagocytes into five distinct subpopulations according to their cell surface markers, including CD11b^hi^ CD11c^hi^, CD11b^hi^ CD11c^lo^, CD11b^int^ CD11c^int^, CD11b^lo^ CD11c^hi^, and CD11b^−^ CD11c^int^. CD11b^int^ CD11c^int^ F4/80^high^ monocytes perform anti-inflammation effects as endogenous defenders. Kidney resident macrophages are *in situ* self-renewal and minimally differentiated from circulating monocytes after ischemic AKI. However, bone marrow-derived monocytes can replenish the kidney resident macrophages when they are depleted using polyinosinic/polycytidylic acid (poly I:C), consistent with the niche competition hypothesis (38). Interestingly, kidney resident macrophages lack major histocompatibility complex class II (MHCII) expression in the repair phase after AKI, a phenotype occurring during the nephrogenesis, and enrich Wnt ligands production, such as Axin2, Tcf4, and Jun (38). In ischemic AKI, C-C chemokine receptor type 2 (CCR2) deficiency alleviates circulating Ly6C^+^ macrophage recruitment and kidney injury and augments interstitial accumulation of Ly6C^-^ embryonic yolk sac-derived resident macrophages and kidney fibrosis in late phases (41). Clodronate Liposome-induced macrophage depletion attenuates kidney injury and fibrosis, which can be restored by adoptive transfer of Ly6C^−^ macrophages from injured wide type kidneys. While Ly6C^−^ macrophage-derived cytokines facilitate the fibroblast-myofibroblast differentiation *in vivo* and *in vitro*, direct evidence targeting the trans-differentiation from Ly6C^−^ macrophages to myofibroblasts remains missing (41). Similarly, CX3CR1^+^ resident renal phagocytes amplify leukocyte infiltration in an NLRP3-dependent manner in contrast-induced acute kidney injury (42). Accumulation of infiltrating and resident macrophages augments in autosomal dominant polycystic kidneys. In unilateral nephrectomy accelerated *Pkd1* mice, lrf5 expression in resident macrophages aggravates cystic disease severity by producing pro-inflammatory cytokines (43). In ischemia-reperfusion injury (IRI) accelerated cystic mice, the phenotype of kidney resident macrophages transfers from F4/80^high^ CD11C^low^ to F4/80^high^ CD11C^high^, and reappearance of juvenile-like resident macrophages correlated with the accelerated cyst formation (44).

However, kidney resident macrophages also perform protective effects during acute and chronic kidney disease. Park et al. (45) found renal repair after ischemic AKI in mice lacking kidney resident CD45^+^ Ly6G^−^ F4/80^high^ CD11b^int^ macrophages but containing infiltrating CD45^+^ Ly6G^−^ F4/80^int^ CD11b^high^ macrophages is delayed compared to the wide type mice. V-domain Ig suppressor of T cell activation (VISTA), an inhibitory immune checkpoint molecule, is mainly expressed by CD45^+^ Ly6G^−^ F4/80^high^ CD11b^int^ kidney resident macrophages and has the biomarker potential in distinguishing the renal macrophages (45). CX3CR1 mediated phagocytes by kidney resident macrophages initiate within the first hours during the innate host defense against *Candidiasis*, confirmed by CX3CR1-M280 associated susceptibility to systemic candidiasis in humans (46). CD11b^int^ F4/80^bright^ kidney resident macrophages protect renal artery stenosis-induced kidney injury by promoting the proangiogenic environments (36). Thus, kidney resident macrophages perform diverse effects depending on the phase and injury types. Based on a better understanding of cell surface markers and *Cre* specificity, further strategies should be explored to maintain protective resident macrophage phenotype during kidney disease. To advance these studies toward clinic interventions in patients, we must overcome two shortages of kidney resident macrophages. Firstly, minimal information is known about the anatomy and functions of resident macrophages in human kidney. Secondly, the similarities and differences of resident macrophages between rodent models and human kidneys remain unclear.

## Monocyte as Precursors of Kidney Macrophages

While circulating monocytes minimally contribute to the renal macrophage pool under homeostasis, toxic or infectious damages result in augmented recruitment of monocyte-derived macrophages into the kidney. When renal macrophage niches are ablated, peripheral monocytes rapidly differentiate and replenish kidney macrophages (Munro et al. Nature Communications 11(1):2280 DOI:10.1038/s41467-020-16158-z). Bone marrow-derived monocyte precursors can reconstitute ischemic kidney macrophages in niches when kidney resident macrophages are depleted (38). Colony-stimulating factor-1 (CSF-1) stimulates macrophage proliferation at various time phases and tissues (47). Genetic deficiency or pharmacological blockade targeting CSF-1 inhibits macrophage proliferation, therefore prolonging the tissue repair phase after AKI (48). Rodent models revealed that Ly6C^high^ pro-inflammatory monocytes infiltrate early in damaged kidneys (49, 50), depending on chemokines such as CCL2, CCL5, and CX3CR1 (26, 51–53). Initial infiltrating bone marrow-derived macrophages are characterized as Ly6C ^high^ iNOS^+^ cells by flow cytometry in rodent kidneys, whereas late Ly6C^low^ macrophages perform profibrotic M2-like effects (49, 50). CD11b diphtheria toxin receptor (DTR)-mediated depletion of monocyte/macrophages (50) or pharmacological blockade targeting chemokine pathways (54) attenuates kidney fibrosis, suggesting a profibrotic role of infiltrating macrophages in renal fibrogenesis. In severe IRI-induced AKI-CKD models, adoptive transplantation of F4/80^int^ B7-H4^high^ (M2c) macrophages rather than F4/80^high^ B7-H4 ^int^ (M1) macrophages restore renal interstitial fibrosis in liposome clodronate-induced macrophage abrogated mice (55). Similarly, adoptive transfer of F4/80^+^ CD301^+^ (M2) macrophages rescue renal fibrosis in obstructed kidneys after macrophage depletion (56). Monocyte-derived kidney macrophages aggravate fibroblast activation and renal fibrosis by secreting cytokines (1, 57). Despite the direct and indirect profibrotic effects, bone marrow-derived macrophages can transdifferentiate into collagen-producing myofibroblasts *via* macrophage-myofibroblast transition (MMT) (58). Using Lyz2-Cre/Rosa26-Tomato mice, lineage tracing studies demonstrated that approximal 50% of the αSMA^+^ Collagen^+^ myofibroblasts derive from F4/80^+^ Tomato^+^ myeloid cells (59, 60). In contrast, a 2018 study challenges the MMT hypothesis as bone marrow-derived myofibroblasts make a limited contribution to the myofibroblasts in the obstructed kidney (61). The conflicting results come from the identification of myeloid cells by CD45^+^ and myofibroblasts by PDGFRβ^+^, as unspecific markers amplify the miscalculation. Another limitation is the deficiency of markers to distinguish bone marrow-derived fibroblasts from macrophage-derived myofibroblasts.

Moreover, macrophages perform diversified roles in renal fibrogenesis *via* secreting matrix metalloproteinases. Matrix metalloproteinases, especially macrophage-derived matrix metalloproteinase-9 (MMP-9), promote kidney fibrosis through stimulating extracellular matrix deposition (62, 63). In contrast, Twist1 in infiltrating macrophages promotes extracellular matrix degradation by stimulating CD11b^+^ Ly6C^low^-derived matrix metalloproteinase-13 (MMP-13) production (64). As CD11b^+^ Ly6C^high^ monocytes freshly infiltrate after kidney injury and represent the onset of renal inflammation, CD11b^+^ Ly6C^int^ and CD11b^+^ Ly6C^low^ populations expand in the phases of repair and fibrosis (41). The evidence that terminally differentiated macrophages rather than freshly infiltrating monocyte progenitors are the major players in kidney fibrogenesis suggests that kidney macrophages function varies due to disease types and time phases.

The Ly6C^low^ subpopulation of circulating monocytes is characterized by monitoring and phagocytosing circulating immune complexes (65). These Ly6C^low^ monocytes present antigens and activate effector CD4 ^+^ T lymphocytes in the glomerular capillaries (66). Circulating myeloid-derived suppressor cells (MDSCs) are immune suppressive populations and initially investigated in cancer (67). MDSCs were firstly reported to maintain cardiac transplant tolerance in rodent models (46), whereas renal MDSCs accumulation positively correlates with graft survival and kidney transplant recipients (68, 69). The effects of MDSCs further expand to immune-regulation and fibrogenesis during septic and metabolic kidney disease (70, 71), suggesting the therapeutic potential of MDSCs in acute and chronic kidney disease.

## Heterogeneity of Human Kidney Macrophages and Clinical Transformation

Human monocyte/macrophages are classified into three distinct populations, including classical CD14^++^ CD16^−^ subset, non-classical CD14^+^ CD16^+^ subset, and intermediate CD14^++^ CD16^+^ subset (72). In a 35 months cohort with 94 dialysis patients, the number of classical CD14^++^ CD16^−^ monocytes can independently predict cardiovascular events and death (73). Rogacev et al. (74) demonstrated CD14^++^ CD16^–^ monocytes numbers can also predict cardiovascular events in CKD patients. Non-classical CD14^+^ CD16^+^ monocytes from CKD patients express high levels of chemokines, facilitating their adhesion to vascular walls (75). Pro-inflammatory CD14^+^ CD16^+^ monocytes correlate with blood vessel stiffness in predialysis CKD patients, suggesting that non-classical CD14^+^ CD16^+^ subset damage endothelial cells (76). However, most clinical studies lack direct evidence of macrophage populations inside the kidney, and the predictive ability of human macrophage populations in kidney injury and fibrosis requires further investigations.

To expand experimental knowledge of macrophages to clinical applications, we still have to overcome several obstacles. Firstly, available data of human kidney macrophages is significantly insufficient, especially the dynamic changes of macrophage function and subpopulations. Secondly, CD14^++^CD16^−^ and CD14^++^CD16^+^ monocytes resemble mouse Ly6C^+^ inflammatory monocytes, whereas CD14^+^CD16^+^ monocytes share phenotypic features with Ly6C^-^ anti-inflammatory monocytes and adhering vascular endothelium (77). As classical CD14^++^ CD16^–^ subset is known for the phagocytic capability, the overlapping and sometimes conflicting features of human and murine monocytes require further investigation. Thirdly, the functionality of kidney macrophages is dynamically variable and affected by the nature of kidney diseases. Thus, clinic translation must address the characteristics of kidney macrophages in different types and phases of the disease (**Table 1**).

**Table 1:** Macrophage markers in human and mouse.

Markers	Gene	Species	Protein Type	Reference
B7-H4	VTCN1	Human, Mouse	Cell Membrane Receptor	(55)
B7-H5 (VISTA)	VSIR	Human, Mouse	Cell Membrane Cytokine	(45)
CD11b	ITGAM	Human, Mouse	Cell Membrane Receptor	(30, 36, 41, 45, 64)
CDllc	ITGAX	Human, Mouse	Cell Membrane Receptor	(18, 44, 78)
CDI4	CD I4	Human, Mouse	Cell Membrane Receptor	(72–76)
CD16	FCGR3A	Human, Mouse	Cell Membrane Receptor	(72–77)
CD45	PTPRC	Human, Mouse	Cell Membrane Receptor	(30, 45)
CD115	CSF1R	Human, Mouse	Cell Membrane Receptor	(30)
CD192	CCR2	Human, Mouse	Cell Membrane Receptor	(79)
CD206	MRC1	Human, Mouse	Cell Membrane Receptor	(13, 80)
CCRL1	CX3CR1	Human, Mouse	Cell Membrane Receptor	(37, 42)
F4/80	ADGRE1	Human, Mouse	Cell Membrane Receptor	(13, 18, 30, 36, 40, 44, 45, 55, 56, 81, 82)
iNOS	NOS2	Human, Mouse	Cytoplasm Enzyme	(49, 50)
Ly6C	Ly6c1	Mouse	Cell Membrane Receptor	(30, 41, 49, 50, 77)
MHC class II	MHCII	Human Mouse	Cell Membrane Receptor	(38)
MCP-1	CCL2	Human Mouse	Secreted Cytokine	(26, 79)
SCARD1	CD68	Human Mouse	Cell Membrane Receptor	(83)
TNF-α	TFN	Human, Mouse	Cell Membrane Cytokine	(57)

Nevertheless, characterized macrophage recruitment and maturation pathways are also appliable in patients with kidney diseases. Renoprotective effects of interventions targeting CCL2/CCR2 have been confirmed in rodent models of kidney inflammation and fibrosis (26, 41). Consistently, CCR2 inhibitor CCX140-B further attenuates albuminuria levels in patients with type 2 diabetes in addition to the standard renin-angiotensin system (RAS) blocking therapies (84). Although the definite effects of CSF1R in macrophage recruitment and proliferation, the role of CSF1R inhibitors and neutralizing antibodies have not been tested in clinical trials to treat kidney diseases. The JAK-STAT pathway regulates macrophage activation and renal function decline in patients with type 2 diabetic nephropathy (85). JAK1 and JAK2 inhibition by Bariticinib decreases albuminuria levels in patients with diabetic kidney diseases (DKD) (86). Our recent studies found that macrophage-derived extracellular vesicles are kidney-targeted drug carriers and worth further expansion into clinical trials (87).

## Single-Cell RNA-Sequencing Revolution

After the qualitative leap from the oversimplified M1/M2 paradigm to individual subpopulations identified by cell surface markers, single-cell RNA-sequencing (scRNA-seq) categorizes macrophages by their function and phenotype, revealing the continuum and complexity of macrophages during the development and pathogenesis of kidney disease (88, 89). Zimmerman et al. (80) identified *C1q* expression as a novel marker of resident macrophage clusters in mouse kidneys, and *C1q* expressing clusters in other species were verified by the expression of *Cd74*, *Cd81*, and *Apoe*. In a rodent model of rhabdomyolysis-induced AKI, F4/80^low^ CD11b^high^ Ly6b^high^ CD206^low^ pro-inflammatory macrophages infiltrate early after rhabdomyolysis, while F4/80^high^ CD11b^+^ Ly6b^low^ CD206^high^ reparative macrophages are dominant at late phase. However, the scRNA-seq analysis demonstrated that signal pathways do not precisely match macrophage phenotypes and the existence of individual subpopulations simultaneously expressing heterogeneous markers (81). At the late phase after ischemic AKI, macrophages expressing *Ccl2* and *Ccr2* infiltrate around the Vcam1^+^ damaged tubules (79). By evaluating the typical *C1qa*, *Cd74*, and *Adgre1* expression, macrophages are defined as the predominant immune cells in diabetic glomeruli and mainly M1-like macrophages (90). The scRNA-seq analysis revealed that both extent and levels of *Axl* expression increased in F4/80^+^ macrophages from rejecting allografts compared to tolerized kidneys; and *Axl* promotes the differentiation of intra-graft myeloid cells towards pro-inflammatory phenotypes after transplantation (82). Moreover, the combination of scRNA-seq and lineage tracing technique attracts particular attention. Lineage tracing can elucidate the clonal relationships during development and differentiation, enable lifecycle monitoring. In contrast, scRNA-seq can identify exact cell types but unable to determine the lineage relationships. Thus, integrating scRNA-seq and lineage tracing will provide extra information about cell types, development, and differentiation in a longstanding pattern.

Stewart et al. (78) demonstrated that human kidney monocyte phagocytes (MNPs) expressing *ITGAX* and *HLA-DRA* are categorized into four distinct clusters (MNPa to MNPd). MNPa subpopulation expressing *CD14* in mature kidney analogous classical monocytes, whereas MNPb expressing *CD16* is transcriptionally similar to non-classical monocytes. Consistently, CD14^+^ CD68^+^ monocyte/macrophages are the most abundant immune cells in urine and account for one-third of urinary clusters. These monocyte/macrophages are enriched in genes related to antigen presentation and macrophage activation and further classified into CD16^+^ and CD16^-^ subpopulations (83).

## Conclusions and Perspectives

**Figure 1:**
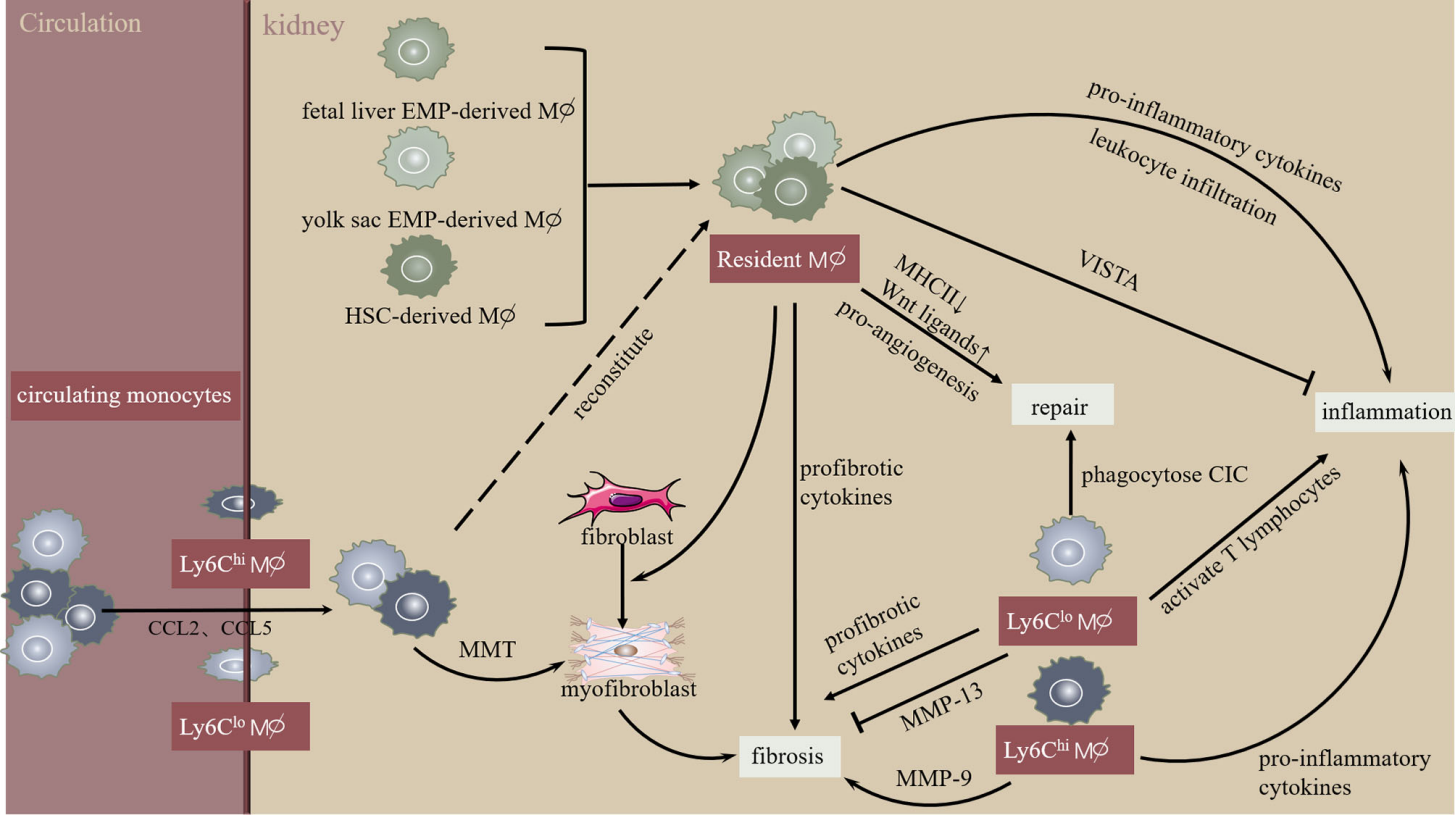
Macrophage heterogeneity during initiation and progression of kidney injury and fibrosis. Kidney resident macrophages derive from multi-sources and monitor trans-endothelial transport of circulating immune complexes. In the initial phase of kidney injury, resident macrophages stimulate leukocyte infiltration and cytokine secretion. Interestingly, kidney resident macrophages express V-domain Ig suppressor of T cell activation (VISTA), an inhibitory immune checkpoint molecule. Increased chemokines (CCL2, CCL5) promote circulating monocyte chemotaxis into the kidney, developing into infiltrating Ly-6C^hi^ macrophages exhibiting pro-inflammatory phenotype or macrophage-myofibroblast transition (MMT). Similarly, infiltrating Ly6C^low^ macrophages promote kidney inflammation and fibrosis *via* activating T lymphocytes or pro-inflammatory cytokines. However, Ly6C^hi^ macrophages inhibit kidney fibrosis by producing MMP-13. Overall, these mechanisms lead to extracellular matrix dynamic homeostasis during the resolution of kidney injury and fibrosis.

Macrophages are recruited by chemokines and contribute to the pathogenesis of kidney injury, repair, and fibrosis. Despite the wide application of the oversimplified pro-inflammatory M1 and anti-inflammatory M2 macrophage paradigm, macrophage complexity in origin, phenotype, and function has attracted attention. Macrophage subpopulations were distinguished by combining cell surface markers and using novel single-cell RNA sequence technology to explore macrophage contribution in tissue injury, regeneration, and fibrosis. Our data and others have confirmed the therapeutic potential of macrophage pathways in acute and chronic kidney diseases; however, the functionally opposing macrophage subpopulations require incisive and tissue-specific strategies (**Figure 1**). Moreover, an in-depth understanding of the specialty and commonality in scRNA-seq defined macrophage clusters requires further investigation.

## Author Contributions

YW and H-RY contributed equally to the writing of the manuscript. BW contributed to the figure of the manuscript. B-CL conceived the concept and contributed to the writing of the manuscript. All authors contributed to the article and approved the submitted version.

## Funding

This work was supported by China National Natural Science Foundation (8203000544, 81720108007) and the key research project of the Ministry of China Science and Technology (2018YFC1314000) to B-CL as PI, and National Natural Science Foundation of China (81900623), and the Natural Science Foundation of Jiangsu Province (BK20190349) to YW as PI.

## Conflict of Interest

The authors declare that the research was conducted in the absence of any commercial or financial relationships that could be construed as a potential conflict of interest.
